# The precise function of alphaherpesvirus tegument proteins and their interactions during the viral life cycle

**DOI:** 10.3389/fmicb.2024.1431672

**Published:** 2024-07-02

**Authors:** Yuxi Cui, Mingshu Wang, Anchun Cheng, Wei Zhang, Qiao Yang, Bin Tian, Xumin Ou, Juan Huang, Ying Wu, Shaqiu Zhang, Di Sun, Yu He, Xinxin Zhao, Zhen Wu, Dekang Zhu, Renyong Jia, Shun Chen, Mafeng Liu

**Affiliations:** ^1^Engineering Research Center of Southwest Animal Disease Prevention and Control Technology, Ministry of Education of the People’s Republic of China, Chengdu, China; ^2^Key Laboratory of Animal Disease and Human Health of Sichuan Province, Chengdu, China; ^3^International Joint Research Center for Animal Disease Prevention and Control of Sichuan Province, Chengdu, China; ^4^Institute of Veterinary Medicine and Immunology, Sichuan Agricultural University, Chengdu, China; ^5^Research Center of Avian Disease, College of Veterinary Medicine, Sichuan Agricultural University, Chengdu, China; ^6^Sinopharm Yangzhou VAC Biological Engineering Co., Ltd., Yangzhou, China

**Keywords:** alphaherpesvirus, tegument protein, life cycle, interaction, innate immune escape, autophagy, ESCRT

## Abstract

Alphaherpesvirus is a widespread pathogen that causes diverse diseases in humans and animals and can severely damage host health. Alphaherpesvirus particles comprise a DNA core, capsid, tegument and envelope; the tegument is located between the nuclear capsid and envelope. According to biochemical and proteomic analyses of alphaherpesvirus particles, the tegument contains at least 24 viral proteins and plays an important role in the alphaherpesvirus life cycle. This article reviews the important role of tegument proteins and their interactions during the viral life cycle to provide a reference and inspiration for understanding alphaherpesvirus infection pathogenesis and identifying new antiviral strategies.

## 1 Introduction

Herpesviruses are a class of double-stranded DNA viruses with a tegument structure (Davison et al., 2009). According to the different biological characteristics and genetic structures of viruses, the International Committee on Taxonomy of Viruses (ICTV) has divided *Herpesviridae* into three subfamilies: *Alphaherpesvirinae, Betaherpesvirinae, and Gammaherpesvirinae* (Gatherer et al., 2021). At present, ICTV has classified *Alphaherpesvirinae* a into 49 viruses in 5 genera, common type includes herpes simplex virus type-1/2 (HSV-1/2), duck plague virus (DPV), pseudorabies virus (PRV), varicella-zoster virus (VZV), equine herpesvirus (EHV), bovine herpesvirus (BHV-1) and Marek’s disease virus (MDV)and so on (International Committee on Taxonomy of Viruses Executive Committee, 2020).

The complete virion of all herpesviruses consists of four parts: the core containing the double-stranded DNA genome, the capsid, the tegument and the envelope. The presence of a complex tegument between the viral capsid and the envelope is typical of herpesvirus morphology, and this layer contains many proteins (Table 1), which can be divided into three groups: inner tegument proteins closely related to the nucleocapsid, outer tegument proteins that contact the capsule membrane and “intermediate” tegument proteins, which are unique to the *Alphaherpesvirinae*. Intermediate tegument proteins are among the most abundant tegument proteins and act as the central hub of the protein interaction network (Wolfstein et al., 2006; Radtke et al., 2010).

**TABLE 1:** Main functions of alphaherpesvirus tegument proteins.

Tegument protein	Main functions	References
pUL7/ ORF53	Virus assembly and cell-to-cell spreading, stabilize focal adhesions and maintain cell morphology	Albecka et al., 2017; Feutz et al., 2019; Butt et al., 2020
pUL11/ ORF49	Secondary envelopment, membrane fusion	Kim et al., 2013; Yang et al., 2021; Yang et al., 2022
pUL13(VP18.8)/ ORF47	Protein kinase, tegument dissociation, regulate the NEC, assembly and release, regulates apoptosis, inhibits the innate immunity, sustained long distance axon transport	Kato et al., 2006; Coller and Smith, 2008; Jarosinski and Osterrieder, 2010; Gershburg et al., 2015; Koyanagi et al., 2018; Pennisi et al., 2021; Zhao et al., 2022
pUL14/ ORF46	Nuclear transport of VP16 and viral capsids, regulates apoptosis, viral replication	Yamauchi et al., 2008; Oda et al., 2016; Wan et al., 2022
pUL16/ ORF44	Nuclear egress of capsids, secondary envelopment, promotes the metabolism of cell mitochondria, membrane fusion	Chadha et al., 2017; Gao et al., 2017; Gao et al., 2020; Li et al., 2021
pUL21/ ORF38	Nuclear egress of capsids, inhibits the innate immunity, secondary envelopment and cell-to-cell spreading, regulates microtubule assembly, membrane fusion	Sarfo et al., 2017; Shahin et al., 2017; Yan et al., 2019; Gao et al., 2020; Muradov et al., 2021; Ma et al., 2023
pUL23/ ORF36/ TK	Thymidine kinase	Yin et al., 2020
pUL36(VP1/2)/ ORF22	Capsid transport, inhibits the innate immunity, secondary envelopment	Fan et al., 2015; Ye et al., 2017; Musarrat et al., 2021; Mohnke et al., 2022
pUL37/ ORF21	Capsid transport, secondary envelopment, neuroinvasion, inhibits the innate immunity	Jambunathan et al., 2014; Richards et al., 2017; Zhang et al., 2018; Kim et al., 2024
pUL41(VHS)/ ORF17	Induces host shutoff, inhibits the innate immunity, neurovirulence	Black et al., 2014; Ma et al., 2019; He et al., 2021; Yan et al., 2024
pUL46(VP11/ 12)	Inhibits the innate immunity, secondary envelopment	Taddeo and Roizman, 2006
pUL47(VP13/ 14) / VP8/ ORF11	Inhibits the innate immunity, regulates VHS and pUL48-dependent transcription, secondary envelopment, induces apoptosis	Liu et al., 2014; Afroz et al., 2018; Chuard et al., 2020; He et al., 2020
pUL48(VP16) / ORF10	Activate the transcription of viral immediate-early genes, viral DNA replication, virus assembly	Fuchs et al., 2002a; Fan et al., 2020
pUL49(VP22) / ORF9	Secondary envelopment, regulates VHS, regulates translocation of viral and cellular proteins, promotes neurovirulence, promotes protein synthesis, accumulated viral mRNA	Duffy et al., 2009; Mbong et al., 2012a; Tanaka et al., 2012; Rémy et al., 2013; Trapp-Fragnet et al., 2019; Xu et al., 2021; Wu et al., 2023
pUL50(dUTPase) / ORF8	DUTPase, viral DNA replication, inhibits the innate immunity	Fuchs et al., 2000; Zhang et al., 2017
pUL51/ ORF7	Secondary envelopment and cell-to-cell spreading, viral replication and pathogenicity	Kato et al., 2018; Feutz et al., 2019; Butt et al., 2020
pUL55/ ORF3	Unknown	
pUS2	Inhibits the innate immunity	Lu et al., 2017
pUS3/ ORF66	Protein kinase, tegument dissociation, inhibits the innate immunity, regulates VHS	Deng et al., 2020; Muradov et al., 2021; Roller and Johnson, 2021; Du et al., 2022; van Gent et al., 2022; Zhou et al., 2024
pUS10/ ORF64	Unknown	
pUS11	Regulates host translation, capsid transport, inhibits the innate immunity and autophagy,	Lussignol et al., 2013; Kew et al., 2016; Liu et al., 2019; Musarra-Pizzo et al., 2022
ICP0(RL2) / ORF61/ EP0	Inhibits the innate immunity, E3 ubiquitin ligase	El-Mayet et al., 2021; Zhang et al., 2021; Parameswaran et al., 2024
ICP4(RS1)/ ORF62/ IE180	Regulates transcription	Dunn and Baines, 2023
ICP34.5(RL1)	Prevents host translational shutoff and autophagy	Tang et al., 2019; Ripa et al., 2022

Herpesviruses are dependent on host cells for proliferation, and the virus enters host cells through endocytosis and membrane fusion (Mercer et al., 2010). When virions infect cells, viral glycoproteins mediate the fusion of the viral envelope and cell membrane (Johnson and Baines, 2011). Subsequently, the viral nucleocapsid is released into the cytoplasm, and the viral DNA migrates to the nucleus through the nuclear pore and begins to be replicated, initiating transcription of the viral genome (Liashkovich et al., 2011). The capsid assembles in the nucleus, and the genome is packaged into it (Heming et al., 2017). Next, the virions encased in the primary envelope enter the inner nuclear membrane (INM) through budding and then fuse with the outer nuclear membrane to undergo de-envelopment (Bigalke and Heldwein, 2016). The viral capsid is released into the cytosol, where the virus then acquires the tegument and the secondary envelope to reach maturation, and the progeny virus is discharged from the cell by exocytosis or via the endoplasmic reticulum system (Crump, 2018).

Tegument proteins and their interactions play important roles in the viral life cycle, including but not limited to targeting the movement of virion components into and out of the nucleus, recruitment of molecular motors during movement into and out of the cell, regulation of viral and host gene and protein expression, and assembly of virions during nuclear egress (Kelly et al., 2009). They also provide a scaffold for the assembly of the virion, creating a network of interactions that connect the capsid and the viral envelope proteins (Guo et al., 2010). This article reviews the important functions of tegument proteins and their interactions at various stages of the viral life cycle, with an emphasis on tegument proteins from the *Alphaherpesvirinae*, in particular HSV-1 PRV and VZV, since these represent the most extensively studied.

## 2 Function of alphaherpesvirus tegument proteins at various stages of the virus life cycle

### 2.1 Virus adsorption, invasion and decapsidation

Herpesvirus attachment to cell surfaces is a relatively nonspecific process based on electrical charge and is achieved by reversible binding of viral envelope glycoproteins (gC and gB in *Alphaherpesvirinae*) to heparan sulfate proteoglyca(HSPG) and chondroitin sulfate proteoglycan(CSPG) (Spear and Longnecker, 2003). These initial entry steps rely on interactions between glycoproteins, primarily gB, gC, and gD, as well as host cell receptors and do not directly require tegument proteins (Spear, 2004). The virus particles entering the cell undergo different degrees of degeneration, expansion, and cleavage, and some virus components are released; subsequently, most of the viral tegument proteins are dissociated, and their respective functions are then exerted. This dissociation process occurs mainly via the viral kinases pUS3 and pUL13 (Morrison et al., 1998). The remaining tegument proteins are connected to the nucleocapsid and participate in the transport of microtubule-dependent cytokinetic proteins to the nucleocapsid. The HSV-1 inner tegument proteins pUL36 and pUL37 interact with the cellular motor proteins kinesin and dynein, respectively, and these interactions lead to bidirectional or hopping movement of the capsid along the microtubule (Döhner et al., 2002; Douglas et al., 2004; Antinone and Smith, 2010; Radtke et al., 2010; Kramer and Enquist, 2013). For PRV, VP1/2 (pUL36 homologous protein) interacts with the Dynein intermediate chain (DIC) and the large subunit p150 of the dynactin-activating protein to transport cargo through a network of microtubules (Bremner et al., 2009). However, in HSV-1, the pUL37 protein interacts with dynetin DIC during the early stage of infection. This pUL37-DIC interaction is synchronized with DIC phosphorylation in infected cells (Musarrat et al., 2021). Since the kinetic protein and kinetic protein motor complex cooperate in retrocellular transport, the binding of pUL37 to the kinetic protein may stabilize the movement of the entire virus, facilitating capsid transport to the nucleus within the cell. In addition, pUL34 can interact with DYNC1/1a (Ye et al., 2000; Martínez-Moreno et al., 2003). The inner tegument proteins participate in the recruitment of molecular motors, thus enabling transport of the viral capsid along microtubules.

In this process, major structural components of the HSV-1 tegument are phosphorylated upon entry into infected cells; for example, the phosphorylation of VP13/14 and VP22 mediates their dissociation from virions *in vitro*, and the phosphorylation of VP1/2 may be necessary for its interaction with the remaining tegument (Morrison et al., 1998).

### 2.2 Viral gene transcription and DNA replication

The viral DNA entering the nucleus is cycled to stimulate the transcription and translation of the viral immediate early (IE) gene under the action of the transcriptional activator VP16, and after translation, the IE product activates and regulates the transcription of the early (E) gene. The translated E protein products activate and regulate the transcription of late (L) genes, which are mainly structural proteins, in a cascade reaction (Simmen et al., 1997; Liu et al., 2015). Therefore, the tegument protein VP16 is a key promoter of this process. VP16 works with host cytokine 1 (HCF-1) and octamer transcription factor-1 (Oct-1/POU2F1) to form a complex (VIC) that activates transcription of the immediate early rise gene and is critical for initiating the lytic replication of HSV-1 (Shaw et al., 1995). After the entry of HSV-1 into cells, HCF-1 interacts with VP16 through six Kelch domains on its N-terminus (amino acid sequence 3-455 aa) and mediates the nuclear input of VP16 (Shaw et al., 1995). After entering the nucleus, the host protein Oct-1 binds to the HFC-1 and VP16 complexes via its POU-specific domain (POUS) and recognizes five HSV-1 IE genes (ICP0, ICP4, ICP22, ICP27, ICP27). The VP16 reaction sequence (TAATGARAT) in the promoter activates its transcription by recruiting lysine-specific demethylase 1 (LSD1) to demethylate the histone bound to the IE promoter (Liang et al., 2009; Zhou et al., 2010; Roizman and Whitley, 2013). Transcription of the IE gene then activates the remaining viral gene, initiating the viral cleavage replication cycle. HSV-1 gene promoters include binding sites for host cell and viral proteins. These sequences include multiple GC-rich sequences or GC boxes in the promoters of the IE and E genes that bind to the cellular transcription factor specific protein 1(Sp1) (Jones and Tjian, 1985; Jones et al., 1985). Sp1 is a universally expressed zinc finger transcription factor with a recognized role in regulating viral and cellular gene transcription (Kaczynski et al., 2003). It can promote HSV-1 transcription in conditions or contexts in which the activity or expression of viral transactivators like VP16 or ICP0 is limited (Sodroski et al., 2024). Following acute infection, BoHV-1 establishes lifelong latency in sensory neurons present in trigeminal ganglia (TG) and unknown cells in pharyngeal tonsil (El-Mayet et al., 2017). In the process, the immediate early transcription unit 1 (IEtu1) promoter drives expression of bICP0 and bICP4, The IEtu1 promoter contains two glucocorticoid receptor (GR) responsive elements (GREs) that are transactivated by activated GR. GC-rich motifs, including consensus binding sites for Sp1, are in the IEtu1 promoter sequences (El-Mayet and Jones, 2024a,b). Members of the KLF family, including Krüppel-like factor 15 (KLF15), belong to the Sp1 transcription factor family (Bieker, 2001; Kaczynski et al., 2003). Members of the KLF family bind GC- or Ca- rich motifs, including some containing consensus Sp1 binding sites (Bieker, 2001). Since BoHV-1 and HSV-1 are GC-rich genomes, KLF15 may stimulate certain viral promoters. Under stress stimulation, GRs and KLF15 form a feed-forward loop that stimulates viral gene expression and productive infection following stressful stimuli. (Mangan and Alon, 2003; Sasse et al., 2013, 2015; El-Mayet et al., 2017). New studies have demonstrated that ICP27 enhancer sequences were transactivated by GR and KLF15. Mutation of a consensus Sp1 binding site within ICP27 enhancer sequences impaired transactivation by GR and KLF15 (Ostler and Jones, 2021).

There are also many tegument proteins that play important roles in this process. In DPV, pUL14 interacts with VP16 via a nonclassical nuclear localization signal (NLS) sequence, facilitating the translocation of VP16 to the nucleus (Li et al., 2016). This process is necessary for effective nuclear transport of VP16 (Yamauchi et al., 2008). VP1/2 (pUL36) is the largest tegument protein of HSV-1, and the interaction between VP16 and VP1/2 induces the transcription of the IE gene (Svobodova et al., 2012). VP16 interacts with the virion host shutoff protein (VHS), which is encoded by the UL41 gene, in this process. Previous studies have shown that during viral replication, HSV-1 VHS induces the accumulation of viral proteins and gradually reduces the production of host proteins (Matis and Kúdelová, 2001). This phenomenon, known as “host shut down”, is achieved through VHS activity, and its structural role is strictly related to endoribonuclease activity, which is responsible for host mRNA degradation and viral mRNA turnover (Smiley et al., 2001). VP16 stimulates IE gene transcription, and VHS inhibits host protein synthesis and induces accelerated turnover of cellular and viral mRNAs, while VHS has been shown to play a role in the transition from IE gene to E gene expression by actively degrading IE gene mRNA (Everly et al., 2002; Esclatine et al., 2004; Taddeo and Roizman, 2006). In this model, another protein, VP22, is also thought to play an important role in transcriptional activation because both VHS and VP22 can bind to VP16 (Smibert et al., 1994; Elliott et al., 1995; O’Regan et al., 2007b); therefore, it has been proposed that these three proteins form the trimer complex needed to neutralize the RNase activity of VHS and that in the absence of VP22 or VP16, VHS eventually degrades viral mRNA in an unrestricted manner, resulting in a complete shutdown of viral protein synthesis (Sciortino et al., 2007; Duffy et al., 2009; Mbong et al., 2012b). In addition, pUL47 can deliver a portion of VHS to the nucleus. In transfected cells, VHS with mutations in the nuclear export signal (NES) steadily degraded mRNA but did not in infected cells, suggesting that pUL47 largely blocked viral mRNA degradation by VHS to help regulate this process (Shu et al., 2013). VP16 triggers the cascade reaction by regulating IE gene transcription, and the subsequent expression of the IE proteins ICP0 and ICP4 can also enhance the effective expression of the HSV-1 gene (Cai and Schaffer, 1992; Mossman and Smiley, 1999). ICP0 is a viral E3 ubiquitin ligase that targets restrictive host cell proteins for degradation and promotes IE, E, and L protein expression (Cai and Schaffer, 1992; Mossman and Smiley, 1999; Boutell et al., 2002; Orzalli et al., 2012). Both VP16 and ICP0 promote the removal of nucleosomes and restrictive heterochromatin deposited on the viral genome, thereby increasing viral DNA accessibility and subsequent gene expression (Hagglund and Roizman, 2004; Herrera and Triezenberg, 2004; Cliffe and Knipe, 2008). ICP4 is a viral transcription factor necessary for the stable expression of the E and L genes (Watson and Clements, 1980; DeLuca and Schaffer, 1985). Moreover, ICP4 can bind to specific or nonspecific sequences in the viral promoter while recruiting universal RNA polymerase II (Pol II) to HSV-1 DNA to increase the transcription of most viral genes (DeLuca and Schaffer, 1985; Sodroski et al., 2024), and VP22 appears to promote ICP4 synthesis after transcription (Okada et al., 2018).

To replicate efficiently, herpesviruses have also evolved numerous sophisticated protective mechanisms. For example, dUTPases are known to catalyze the hydrolysis of dUTP to dUMP and pyrophosphate and to play a role in the accurate replication of DNA genomes by preventing the misincorporation of dUTP into replicated DNA (Sedwick et al., 1986; Vértessy and Tóth, 2009). The HSV-1 dUTPase (vdUTPase) is encoded by the pUL50 gene and is conserved throughout Herpesviridae (Preston and Fisher, 1984; McGeehan et al., 2001). In infected cells, pUS3 phosphorylates serine 187 of dUTPase, and this phosphorylation promotes viral replication by regulating the optimal enzyme activity of dUTPase (Kato et al., 2014c). When serine 187 was replaced by alanine, the virulence of recombinant HSV-1 in the central nervous system of mice was significantly reduced, but it had no effect on viral replication or pathogenicity in the eyes or vagina of mice after ocular or vaginal inoculation, respectively (Kato et al., 2014b). Moreover, this enhancement of vdUTPase activity compensates for the low dUTPase activity in the host cell, resulting in efficient viral replication (Kato et al., 2014a). In addition, HSV-1 ICP34.5 can interact with proliferating nuclear antigens and is also important for regulating viral DNA replication (Brown et al., 1997; Harland et al., 2003); the direct interaction of BHV-1 pUL49 with histones or of HSV-1 pUL49 with TAF-1 appears to regulate nucleosome formation and may play an unknown role in early viral replication (Ren et al., 2001; van Leeuwen et al., 2003).

### 2.3 Nuclear egress and envelopment

The herpesvirus capsid is assembled in the nucleus and undergoes a two-step process to cross the nuclear membrane. First, the capsid becomes encapsulates in the INM with the help of the nuclear egress complex (NEC) protein pUL31/34. Second, the perinuclear envelope virion (PEV) fuses with the outer nuclear membrane (ONM) to transport the capsid into the cytoplasm. Once inside the cytoplasm, the capsid is re-encapsulated in the Golgi/trans-Golgi apparatus, where it produces mature virions (Roller and Johnson, 2021). NEC is a heterodimer complex of pUL31 and pUL34 that is essential for herpesvirus nucleation, where pUL34 acts as a type II membrane protein that tethers the NEC to the INM and exposes pUL31 to the karyoplasm (Mettenleiter et al., 2013). The pUL31 protein of HSV-1 relies on physical interaction with the pUL34 protein for stability in infected cells (Ye et al., 2000), and pUL31 is also necessary for the localization of the pUL34 protein to the nucleus, especially the INM (Yamauchi et al., 2001). During this process, the protein kinase pUS3 is recruited to the thin nuclear layer to phosphorylate and dissociate the NEC, thereby promoting capsid budding and nucleation (Mou et al., 2007, 2009). The phosphorylation of pUL31 and pUL34 determines the nature of nuclear envelope deformation, while the viral protein pUL21 regulates the phosphorylation of the two NEC components (Muradov et al., 2021); pUL47 colocalizes with pUL31, pUL34, and pUS3 in the nuclear membrane and forms high-order complexes with these viral proteins in HSV-1-infected cells. The localization of pUL47, pUL31, and pUL34 on the nuclear membrane is regulated by pUS3 kinase activity (Kato et al., 2011; Deng et al., 2022). Moreover, pUL47 may promote the recruitment of the nucleocapsid by interacting with the pUL17-pUL25-pUL31 complex, thereby upregulating the primary capsular membrane of the nucleocapsid to stabilize the binding between the capsid and the pUL34/pUL31 complex at the nuclear membrane, which is necessary for the formation of an effective primary envelope of the nucleocapsid during HSV-1 nucleation (Liu et al., 2014). In addition to pUS3, pUL13 also acts as a phosphokinase that phosphorylates pUS3 and thus controls the nuclear edge localization of pUL31 and pUL34 (Kato et al., 2006; Cano-Monreal et al., 2009). It has also been suggested that the pUL16 and pUL21 proteins work together to promote capsid nucleation (Gao et al., 2017).

The nucleocapsid buds into the perinuclear space at a specific site in the INM, obtains the primary envelope, fuses with the ENM and is released into the cytoplasm, completing the de-envelopment process. Moreover, the immature virions obtain more tegument proteins (mainly outer tegument proteins) in the cytoplasm and then reach the Golgi apparatus, where secondary envelopment occurs, and the final envelope is obtained by budding (Guo et al., 2010). In the literature, tegument proteins are typically divided conceptually into “inner tegument proteins”, which are more closely associated with the capsid, “outer tegument proteins”, which are more closely associated with the capsule membrane, and “intermediate tegument proteins”, which are located between these two types of proteins (Hogue, 2021). Multiple protein interactions are involved in the process of secondary envelopment. Currently, a widely accepted model is that the viral glycoprotein embedded in the Golgi membrane recruits the outer tegument protein components, while the viral capsid is surrounded by the inner tegument protein (Owen et al., 2015).

VP22 (pUL49) is a central hub of tegument-membrane interactions. VP22 (pUL49) has been reported to bind the cytoplasmic tails of gE, gM, and gD, and BHV-1 VP22 can also interact directly with gN, a glycoprotein encoded by the pUL49.5 gene, without relying on gM (Fuchs et al., 2002b; Elliott et al., 2005; Farnsworth et al., 2007; O’Regan et al., 2007a; Stylianou et al., 2009; Maringer et al., 2012; Pannhorst et al., 2018). VP22 (pUL49) can also bind to pUL16 (Starkey et al., 2014), and pUL16 in turn interacts with pUL21, pUL11, and gE (Farnsworth et al., 2007; Harper et al., 2010; Meckes et al., 2010; Han et al., 2011; Chadha et al., 2012) to provide additional contact with the membrane. Furthermore, VP22 may also bind directly to cell membranes via alkaline residues that bind to acidic cytophospholipids (Brignati et al., 2003). VP16 (pUL48) is another central hub of tegument-membrane interactions and has been reported to bind to gH (Gross et al., 2003), although this interaction may not be conserved (Omar et al., 2013).

In addition, there are several important tegument-membrane interactions, such as those involving pUL51 and pUL11, which appear to be conserved, and they can directly bind the membrane by acyl modification [pUL51 is palmitoacylated (Nozawa et al., 2003); additionally, pUL11 is both myristoylated and palmitoylated (MacLean et al., 1989; Loomis et al., 2001)]. Because the “intermediate tegument protein” type is specific to the alphaherpesviruses, the above membrane contact model involving gD and gE (Roller and Johnson, 2021) is also specific to the alphaherpesvirus subfamily, indicating that the modification of pUL51 and pUL11 is important for f virion release among the entire herpesvirus family.

Furthermore, VP22 and VP16 are also at the core of the tegument protein interaction network. The correct assembly of ICP0 (Maringer and Elliott, 2010), ICP4 (Elliott et al., 2005), and pUL47 (Che et al., 2013; Sucharita et al., 2021) into virions requires the participation of VP22; VP22 is also required for the effective redistribution of VP16, ICP0, ICP4, ICP27, and the cellular protein Hsc-70 into the cytoplasm of infected cells, and the double-leucine motif of VP22 is critical for the regulation of this process (Tanaka et al., 2012). VP22 assembly itself involves two determinants, the C-terminal domain and the internal VP16 interaction domain, both of which are necessary for VP22 to effectively recruit to the virus assembly site (Hafezi et al., 2005), but this regulation is independent of the interaction between the two (O’Regan et al., 2007b). Similarly, pUL16 also facilitates the assembly of VP22, and VP22 assembly is even more dependent on pUL16 than on the other binding proteins, gE and VP16 (O’Regan et al., 2007b). In HSV-1, pUL16, pUL11, and pUL21 form a complex. pUL16 binds to the capsid before it reaches the Golgi apparatus, promoting capsid maturation. pUL11 binds to the nuclear membrane and binds to pUL16, thereby increasing the chances of pUL16 binding to the capsid, and in the absence of pUL11, the ability of pUL16 to bind to the capsid is reduced by 70% (Meckes et al., 2010). Then, pUL11 accumulates in the Golgi apparatus. After the nucleocapsid is successfully transported to the Golgi apparatus, pUL11 and pUL21 are involved in the germination and maturation of virions through interactions (Loomis et al., 2006; Meckes et al., 2010). Finally, pUL11 is involved in the release of the virus into the extracellular environment via direct contact of pUL11 with the membrane; similarly, in the absence of the UL11 gene, empty capsids and DNA-containing capsids accumulate on the nuclear membrane, and infected viruses are rarely released (Baines et al., 1995) In PRV, pUL21 is required for the effective incorporation of pUL46, pUL49 and pUS3 into mature virions (Roller and Johnson, 2021).

pUL36 (VP1/2) is necessary for the successful assembly of VP16 onto the capsid (Ko et al., 2010); pUL36, in turn, interacts with pUL37, and both require each other to be incorporated into the virus particles (Trus et al., 1992; Fossum et al., 2009; Krautwald et al., 2009; Kelly et al., 2012). The pUL37-targeted secondary envelopment of HSV-1 requires pUL36 but does not require a capsid (Desai et al., 2001). This finding can be explained by the interaction of pUL37 with pUL46 and pUL36 with VP16 (Trus et al., 1992; Steiner et al., 2007; Krautwald et al., 2009) because these outer tegument proteins accumulate at the site of secondary envelopment. The N-terminal region of HSV-1 pUL36 has the binding sites of pUL37 and VP16, and the C-terminal region has two binding regions of the outer capsid protein pUL25. Thus, HSV-1 pUL36 serves as a linker between the capsid and other tegument proteins (Cohen et al., 1980; Kelly et al., 2012). The absence of UL36 or pUL37 prevents the acquisition of a large amount of tegument in the cytoplasm, leading to the accumulation of uncoated capsids in the cytoplasm.

pUL51, pUL7, and pUL14 are also conserved in alphaherpesviruses, and the loss of these proteins causes misassembly of the secondary envelope (Nozawa et al., 2002; Fuchs et al., 2005; Klupp et al., 2005; Albecka et al., 2017). Both pUL7 and pUL14 can bind pUL51, possibly forming a three-part complex. During HSV-1 infection, pUL51 forms a complex with pUL7 and is primarily responsible for recruiting pUL7 to the cytoplasmic membrane and the tegument of the virion. In addition, the pUL7-pUL51 complex regulates adhesion stability and maintains cell morphology during infection, but the specific mechanism involved needs further study.

## 3 Other aspects

### 3.1 Tegument proteins are involved in innate immune escape

Because herpesvirus infection is lifelong, the virus must evade the host’s immune response during latent infection to prevent its elimination by the host (Whitley et al., 1998). Most tegument proteins are involved in viral innate immune escape (Yang et al., 2019). For example, the recently reported DPV pUL41 selectively downregulates IRF7 expression by reducing its mRNA accumulation, thereby inhibiting the DNA-sensing pathway (Gao et al., 2022); PRV pUL13 inhibits the expression of RIG-I and MDA5 by inhibiting the activation of the transcription factor NF-κB. pUL13 can interact with the CDN domain of STING and recruit RNF5 to promote its ubiquitination and degradation (Kong et al., 2022). Notably, VP22, one of the major tegument proteins, mediates a different mechanism by which other viral proteins participate in immune escape. VP22 accumulates and effectively destroys preformed cGAS-DNA complexes both *in vitro* and in cells. This inhibition relies on DNA-induced liquid phase separation of viral proteins rather than direct interaction with cGAS (Xu et al., 2021). There are also tegument protein interactions involved in these processes; for example, among antiviral mechanisms, PKR is a typical interferon-stimulated gene (ISG) protein (Duffy et al., 2009; Mbong et al., 2012a). Pennisi et al. (2020) reported for the first time that ph-PKR accumulation occurs in different cell lines via a VHS-dependent mechanism and that a lack of VHS RNAse activity affects the negative regulation of ph-PKR mediated by HSV-1. That is, VHS can affect mRNA accumulation during viral infection, allowing HSV-1 to control PKR. In addition, the authors demonstrated for the first time that both serine/threonine kinase proteins pUS3/pUL13 control ph-PKR accumulation during HSV-1 replication and that pUS3 regulates the phosphorylation of PKR by interacting with VHS (Pennisi et al., 2021).

### 3.2 Tegument proteins regulate autophagy pathways

Autophagy is a typical degradation pathway in which cytoplasmic contents are encapsulated in cell membranes called autophagosomes. Subsequently, autophagosomes can fuse with endosomal organelles to form diplosomes, which can be transported within the cell and fused with lysosomes to form degraded autophagolysosomes (Morishita and Mizushima, 2019; McShane and Selbach, 2022; Zhang, 2022). Uninfected cells typically exhibit low autophagy activity, but autophagy is strongly upregulated in response to cellular stress signals, which may include viral infection processes. Autophagy was originally thought to be antiviral because it can lead to lysosomal degradation of viral proteins and particles (Hogue, 2021). Some herpesviruses have strategies to regulate autophagy, and many rely on cellular autophagy mechanisms to successfully complete their life cycle (Yin et al., 2017; Xu et al., 2018). Examples include pUL21 (Ma et al., 2023)and VP16 (Ming et al., 2022)for PRV and ICP34.5 (Tallóczy et al., 2006; Ripa et al., 2022), pUS11 (Lussignol and Esclatine, 2017) and ICP0 (Liu et al., 2021; Waisner et al., 2023) for HSV-1. ICP34.5 is the first-discovered anti-autophagy viral protein of herpesviruses (Tallóczy et al., 2002). The ability to control autophagy by interacting with BECN1 is necessary for HSV-1 neurovirulence (Orvedahl et al., 2007); pUL21, a recently discovered herpesvirus protein associated with autophagy, triggers cGAS degradation through Tollip-mediated selective autophagy, thereby inhibiting innate immunity (Ma et al., 2023); and the host E3 ligase TRIM23, which induces autophagy to limit HSV-1 replication (and that of additional viruses), triggers proteasomal degradation of ICP0 through ubiquitination of K11 and K48 interactions (Liu et al., 2021). The major autophagy adaptor proteins Sequestosome 1 and Optineurin are downregulated in the early stages of HSV-1 infection (Waisner and Kalamvoki, 2019). In PRV, pUSP14 inhibition promotes the binding of K63-linked polyubiquitin chains in VP16 to K168 and subsequent degradation of VP16 via SQSTM1/p62-mediated selective autophagy (Ming et al., 2022). pUS11 has been shown to protect HeLa cells from heat and astrosporin-induced apoptosis and counteract autophagy responses (Javouhey et al., 2008; Lussignol et al., 2013); it also regulates the cleavage of caspase-8, the initiator of mammalian exogenous or death receptor-mediated apoptosis (Musarra-Pizzo et al., 2022).

However, the genomes of some herpesviruses do not encode the viral proteins ICP34.5 or pUS11, two known autophagy-associated proteins (Heinz et al., 2021); examples include DPV and VZV, although they belong to the same alphaherpesvirus subfamily as HSV-1 and PRV. Whether there is a different pattern in the relationship between DPV or VZV and autophagy remains unclear. However, as mentioned above, most of the viral proteins that have been reported to be involved in the autophagy pathway are tegument proteins, which led us to wonder whether there are other tegument proteins that play a role in the autophagy pathway.

### 3.3 Role of tegument proteins in the ESCRT mechanism

Like many cystal virus families (Votteler and Sundquist, 2013; Hurley, 2015; Scourfield and Martin-Serrano, 2017; Barnes and Wilson, 2019), herpesviruses utilize the ESCRT mechanism required for transport to construct and drive the rupture of their cystal membrane (Pawliczek and Crump, 2009; Kharkwal et al., 2014, 2016; Barnes and Wilson, 2019). The ESCRT machinery manipulates organelle membranes in various normal cellular processes by depositing components of ESCRT-III, a spring-like filament that drives planar bilayer deformation into vesicles and tubules (Alonso et al., 2016; Olmos and Carlton, 2016; Christ et al., 2017; Scourfield and Martin-Serrano, 2017; McCullough et al., 2018). HSV-1 can bypass the normal cellular pathway of ESCRT complex assembly (Pawliczek and Crump, 2009; Christ et al., 2017; Barnes and Wilson, 2019, 2020) by polymerizing ESCRT-III filaments directly on the surface of the cortex-binding capsid to support capsule deformation (Butt et al., 2020). When budding is complete, the ESCRT-III filaments contract their neck membranes to undergo shearing, and this process is accompanied by the disassembly and removal of ESCRT-III via hexameric VPS4 (McCullough et al., 2015, 2018; Christ et al., 2017). There are several tegument proteins involved in linking ESCRT mechanisms. For example, arginine clusters in the HSV-1 pUL34 disordered domain mediate interactions with the linker protein ALIX of ESCRT-III, which results in the recruitment of the INM by the ESCRT-III mechanism for effective primary membrane deposition (Arii et al., 2022). The helix-transhelical conformation of HSV-1 pUL51 is similar to that of the ESCRT-III component CHMP4B, which forms ESCRT-III-like filaments, and pUL51 bends the membrane in the cell, directly promoting membrane shearing during viral assembly. The interaction of pUL51 with pUL7 is conserved in all three herpesvirus subfamilies, and the pUL7 protein regulates the state of pUL51 during this process, controlling its ability to clip the capsule membrane at the correct time (Butt et al., 2020). Mutation of the glutamate 233 residue in the active site of the VPS4 ATPase produces the VPS4-EQ allele, which binds ATP but does not hydrolyze ATP (Sun et al., 2017). Vps4-eq has a major negative effect because hexamers containing a mixture of wild-type VPS4 and VPS4-EQ subunits can bind to ESCRT-III filaments but cannot drive their breakdown, irreversibly “locking” the VPS4 hexamer to the bud neck and preventing cutting (Davies et al., 2010; Han et al., 2015; Sun et al., 2017; Han and Hill, 2019). Thus, the expression of VPS4-EQ blocks the completion of HSV-1 and PRV envelopment (Crump et al., 2007; Kharkwal et al., 2014) and arrests the HSV-1 capsid in a partially membrane-closed state (Crump et al., 2007; Kharkwal et al., 2016). The absence of pUL36 reduces the number of complexes containing the HSV-1 capsid VPS4-EQ by approximately 70% (Barnes and Wilson, 2019), suggesting that pUL36 plays an important role in capsid recruitment to the ESCRT-III/Vps4 deposition site (Kharkwal et al., 2016). However, while pUL36 is necessary for the assembly of multiple outer tegument proteins and capsular proteins into capsular granules (Vittone et al., 2005; Mijatov et al., 2007; Ko et al., 2010; Svobodova et al., 2012; Koenigsberg and Heldwein, 2017, 2018), its role in capside-ESCRt-II-VPS4 assembly may be indirect (Kharkwal et al., 2016). In addition, HSV-1 VP1/2 (pUL36) has been shown to deubiquitinate the ESCRT-I protein TSG101 (Caduco et al., 2013; Calistri et al., 2015).

## 4 Conclusion

**FIGURE 1:**
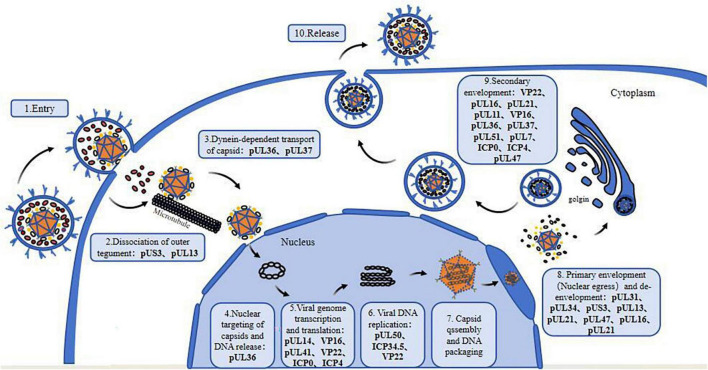
Tegument proteins involved in the life cycle of alphaherpesviruses. 1. Viral entry into the host cell; 2. Dissociation of outer tegument proteins; 3. Dynein-dependent transport of capsid; 4. Nuclear targeting of the capsid and viral DNA release; 5. Transcription and translation of the viral genome; 6. Viral DNA replication; 7. Nucleocapsid formation and viral DNA packaging; 8. Primary envelopment (nuclear egress) and de-envelopment; 9. Secondary envelopment; 10. Maturation and release of virions.

**FIGURE 2:**
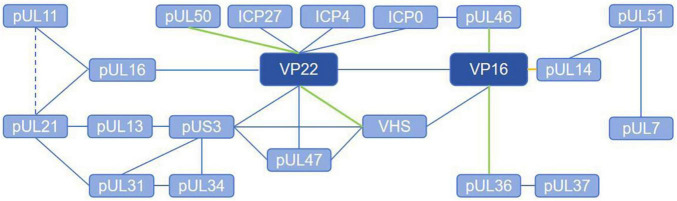
Tegument proteins interaction network of alphaherpesviruses. The solid line represents the direct interaction and the dashed line represents the brief interaction. The blue lines indicate that the interaction is verified with co-ip, the green lines indicate that the interaction is verified with GST-Pulldown, and the yellow lines indicate that the interaction is verified with BiFC.

Herpesvirus tegument proteins play an important role in almost every part of the viral life cycle, especially that of alphaherpesviruses (Figure 1), perhaps because of their unique “intermediate tegument proteins” that are involved in viral particle transport and morphogenesis (Mocarski, 2007). After the virus enters the cell, tegument proteins participate in the dissociation of virus components, nuclear transport of the capsid and docking with the nuclear pore. After the virus enters the nucleus, these proteins regulate mRNA transcription, DNA replication and protein translation. In the later stage of infection, tegument proteins participate in capsid nucleation and primary capsule formation and form extensive and powerful tegument-tegument interactions and tegument-membrane interaction networks during secondary envelopment, which are responsible for virion morphogenesis (Figure 2). It can be assumed that antiviral drugs targeting and blocking conserved tegument protein interactions, such as the interactions between pUL36 and pUL37 or between VP16 and VP22, will be highly important. There is some redundancy in the function of tegument proteins overall, so that particles are still assembled, encapsulated and released from infected cells even after most of the genes encoding structural proteins have been deleted. Because of their redundancy, untangling the tegument protein interactions involved in these complex multistep processes is a challenge, and the use of more advanced techniques, such as liquid chromatography–mass spectrometry (LS-MS), high-resolution confocal microscopy, and bimolecular fluorescence complementarity (BiFC) detection, will help elucidate the protein–protein interactions of tegument proteins in more detail.

Due to the important functions of tegument proteins and their interactions in the viral life cycle, further elucidation of their functions and mechanisms will provide important reference data for the search for therapeutic targets and vaccine development.

## Author contributions

YC: Writing – original draft, Investigation, Methodology. MW: Funding acquisition, Writing – review and editing, Methodology. AC: Funding acquisition, Writing – review and editing, Supervision. WZ: Funding acquisition, Writing – review and editing. QY: Methodology, Writing – review and editing. BT: Conceptualization, Writing – review and editing. XO: Supervision, Writing – review and editing. JH: Methodology, Writing – review and editing. YW: Formal analysis, Writing – review and editing. SZ: Data curation, Writing – review and editing. DS: Resources, Writing – review and editing. YH: Formal analysis, Writing – review and editing. XZ: Supervision, Writing – review and editing. ZW: Formal analysis, Writing – review and editing. DZ: Methodology, Writing – review and editing. RJ: Supervision, Writing – review and editing. SC: Supervision, Writing – review and editing. ML: Methodology, Writing – review and editing.
